# The Role of Superoxide Dismutase 1 in Amyotrophic Lateral Sclerosis: Identification of Signaling Pathways, Regulators, Molecular Interaction Networks, and Biological Functions through Bioinformatics

**DOI:** 10.3390/brainsci13010151

**Published:** 2023-01-15

**Authors:** Sharad Kumar Suthar, Sang-Yoon Lee

**Affiliations:** 1 Neuroscience Research Institute, Gachon University, Incheon 21565, Republic of Korea; 2 Department of Neuroscience, College of Medicine, Gachon University, Incheon 21936, Republic of Korea

**Keywords:** superoxide dismutase 1, antioxidant, amyotrophic lateral sclerosis, canonical pathways, regulators, molecular interaction network, biological functions, toxicity

## Abstract

Mutations in superoxide dismutase 1 (SOD1) result in misfolding and aggregation of the protein, causing neurodegenerative amyotrophic lateral sclerosis (ALS). In recent years, several new SOD1 variants that trigger ALS have been identified, making it increasingly crucial to understand the SOD1 toxicity pathway in ALS. Here we used an integrated bioinformatics approach, including the Ingenuity Pathway Analysis (IPA) tool to analyze signaling pathways, regulators, functions, and network molecules of SOD1 with an emphasis on ALS. IPA toxicity analysis of SOD1 identified superoxide radicals’ degradation, apelin adipocyte, ALS, NRF2-mediated oxidative stress response, and sirtuin signaling as the key signaling pathways, while the toxicity of SOD1 is exerted via mitochondrial swelling and oxidative stress. IPA listed CNR1, APLN, BTG2, MAPK, DRAP1, NFE2L2, SNCA, and CG as the upstream regulators of SOD1. IPA further revealed that mutation in SOD1 results in hereditary disorders, including ALS. The exploration of the relationship between SOD1 and ALS using IPA unveiled SOD1-ALS pathway molecules. The gene ontology (GO) analysis of SOD1-ALS pathway molecules with ShinyGO reaffirmed that SOD1 toxicity results in ALS and neurodegeneration. The GO analysis further identified enriched biological processes, molecular functions, and cellular components for SOD1-ALS pathway molecules. The construction of a protein–protein interaction network of SOD1-ALS pathway molecules using STRING and further analysis of that network with Cytoscape identified ACTB followed by TP53, IL6, CASP3, SOD1, IL1B, APP, APOE, and VEGFA as the major network hubs. Taken together, our study provides insight into the molecular underpinning of SOD1’s toxicity in ALS.

## 1. Introduction

**Figure 1:**
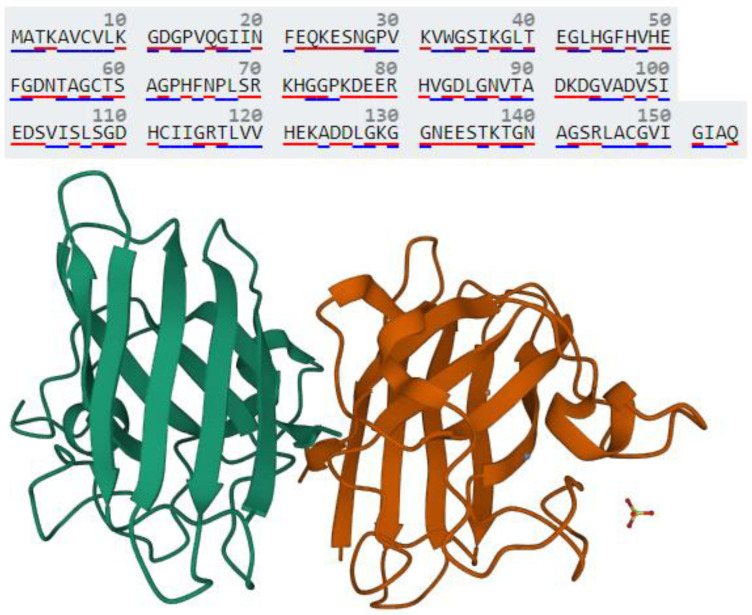
Amino acid sequence and three-dimensional structure of SOD1 (PDB ID: 1PU0). SOD1 is a 32 kDa homodimer protein of 154 amino acids. The red underline of amino acid letters indicates polar residues, while the blue underline indicates hydrophobic residues.

Copper/zinc-binding superoxide dismutase 1 (SOD1) is primarily an antioxidant enzyme present in the cytosol, outer-mitochondrial membrane, and intermembrane space of the mitochondria (Figure 1) [1,2,3]. It detoxifies superoxide radicals (O_2_^•−^) expelled by mitochondria and generated by other redox reactions into molecular oxygen (O_2_) and hydrogen peroxide (H_2_O_2_) [4,5]. As well as acting as an antioxidant, SOD1 via localization from the cytosol to the nucleus combats oxidative stress by regulating the transcription of antioxidant genes involved in cellular defense [6].

Around three decades ago, the identification of the first SOD1 mutation in a group of familial amyotrophic lateral sclerosis (ALS) patients changed the perception of SOD1 being a protective antioxidant to the culprit in ALS [7]. ALS is a neurodegenerative disease characterized by the progressive loss of motor neurons in the spinal cord, leading to spasticity, generalized weakness, muscle atrophy, and paralysis [8,9]. More than 200 different SOD1 mutations have been reported to cause familial ALS [10], accounting for approximately 20% of familial and 2.3% of sporadic ALS [11].

Although wild-type SOD1 is a stable homodimeric protein [12,13,14,15], the mutation in SOD1 decreases the net repulsive charge in the structure, undermining the protein architecture [16]. The destabilized SOD1 mutant in ALS triggers misfolding and aggregation via abnormal disulfide cross-linking, forming toxic inclusions in the mitochondria of neuronal cells [16,17]. The exact mechanism by which SOD1 induces toxicity in ALS remains unknown [18,19].

The number of SOD1 variants in ALS has steadily increased over the last decade (Table S1) [10], while the molecules conveying SOD1 toxicity in ALS remain far from being deciphered, prompting us to investigate SOD1 toxicity in ALS. In this study, we used a set of computational tools, including Ingenuity Pathway Analysis (IPA), STRING, and Cytoscape to analyze the canonical pathways, regulators, functions, and molecular interaction network of SOD1, with an emphasis on SOD1 toxicity in ALS. Our study provides a list of molecules that serve as a bridge between SOD1 and ALS and potentially convey SOD1-imparted toxicity in ALS. The wet-lab researchers can build upon our findings to elucidate the mechanism and pathway of SOD1 toxicity in ALS.

## 2. Materials and Methods

### 2.1. IPA Toxicity Analysis of SOD1

The SOD1 gene (gene ID 6647) obtained as a text file from the National Center for Biotechnology Information (NCBI) [20] database was imported onto the IPA server for new core analysis [21]. The gene identification (GI) number of SOD1 was defined. The “Tox Analysis” function was selected for the new core analysis. The Ingenuity Knowledge Base was used as a reference source. Both direct and indirect relationships with reference to the gene were considered for the predictions. For interaction network generation, 35 molecules per network and 25 networks per analysis were chosen. Along with the interaction network, casual networks were also considered for the predictions. All node types (such as chemicals, complexes, cytokines, diseases, enzymes, functions, etc.) and all data sources were selected for the predictions. For miRNA confidence, only experimentally observed values were considered. Only the human species was considered for predictions. Furthermore, tissues and primary cells, and all types of mutations were selected for the predictions. Right-tailed Fisher’s exact test was used to rank the predictions, where the smaller *p*-value indicates the higher significance of the results. Wherever required the data obtained from IPA were plotted in the Figure formats using GraphPad Prism [22,23].

### 2.2. IPA Exploration of the Pathway/Pathway Molecules between SOD1 and ALS

IPA “genes and chemicals” search of “SOD1” provided the SOD1 gene, which was added to the new pathway to be created between SOD1 and ALS [21]. Similarly, IPA “diseases and functions” search of “amyotrophic lateral sclerosis” provided the list of ALS and associated 631 molecules, which were also added to the pathway to be built between SOD1 and ALS (These 631 molecules were associated with ALS, and not all molecules were associated with SOD1) [21]. To build the path between SOD1 and ALS (predicting molecules connecting SOD1 and ALS), molecules displaying both direct and indirect relationships with SOD1 and ALS were chosen with a direction of relationship from SOD1 to ALS. Ingenuity Knowledge Base was used as a source of molecules and relationships among them. Upon following these steps, IPA identified 87 molecules as nodes between SOD1 and ALS. These node molecules were added to the SOD1-ALS pathway, which resulted in the final SOD1-ALS pathway. The created path was further organized with IPA path designer feature to create the figure for publication. Additionally, the molecule activity predictor (MAP) feature of IPA predicted the effect of increased activity of SOD1 on pathway molecules.

### 2.3. Gene Ontology Analysis of SOD1-ALS Pathway Genes

The ShinyGO v0.76.3 online tool was used for the gene ontology (GO) enrichment analysis of IPA-predicted SOD1-ALS pathway molecules (Table S2; http://bioinformatics.sdstate.edu/go/) [24]. For this, the list of SOD1-ALS pathway molecules identified by IPA was imported on the ShinyGO server and submitted for analysis. ShinyGO first converts all query genes to ENSEMBL gene IDs or STRING-db protein IDs for analysis. Only the human species were selected for analysis. The false discovery rate (FDR) cutoff was set at 0.05 for the predictions. ShinyGO calculates FDR based on the nominal *p*-value from the hypergeometric test.

### 2.4. Construction of Molecular Interaction Network of SOD1-ALS Pathway Molecules

The protein–protein interaction (PPI) network of SOD1-ALS pathway molecules (Table S2) was constructed with STRING v11.5, a tool developed by a consortium of academic organizations (https://string-db.org/cgi/input) [25]. For this, the list of IPA-predicted SOD1-ALS pathway molecules (Table S2) was imported onto the STRING server. For PPI network construction, the “multiple proteins” feature of STRING was used. *Homo sapiens* was selected as the organism for the network construction.

The STRING-constructed PPI network was exported to Cytoscape v3.9.1, an open-source software platform, for further analysis using the STRING option “send network to Cytoscape” (https://cytoscape.org/) [26]. The Cytoscape option “analyze network”, available in the tools category, was used for the analysis of the STRING constructed network. After subjecting the network to analysis in Cytoscape we deselected STRING features of the network such as the glass ball effect, STRING style labels, and STRING style colors. This enabled the network to be designed according to Cytoscape’s features. For the mapping of the PPI network we used the Cytoscape option “style-node-size” followed by column as degree and mapping type as continuous mapping to display the node size corresponding to the degree of the node’s network, while we used the Cytoscape option “style-node-fill color” followed by column as degree and mapping type as continuous mapping to indicate the node color according to the degree of the node’s network.

## 3. Results

### 3.1. Identification of Canonical Pathways, Regulatory Molecules, Biological Functions and Toxicity Outcome of SOD1

**Figure 2:**
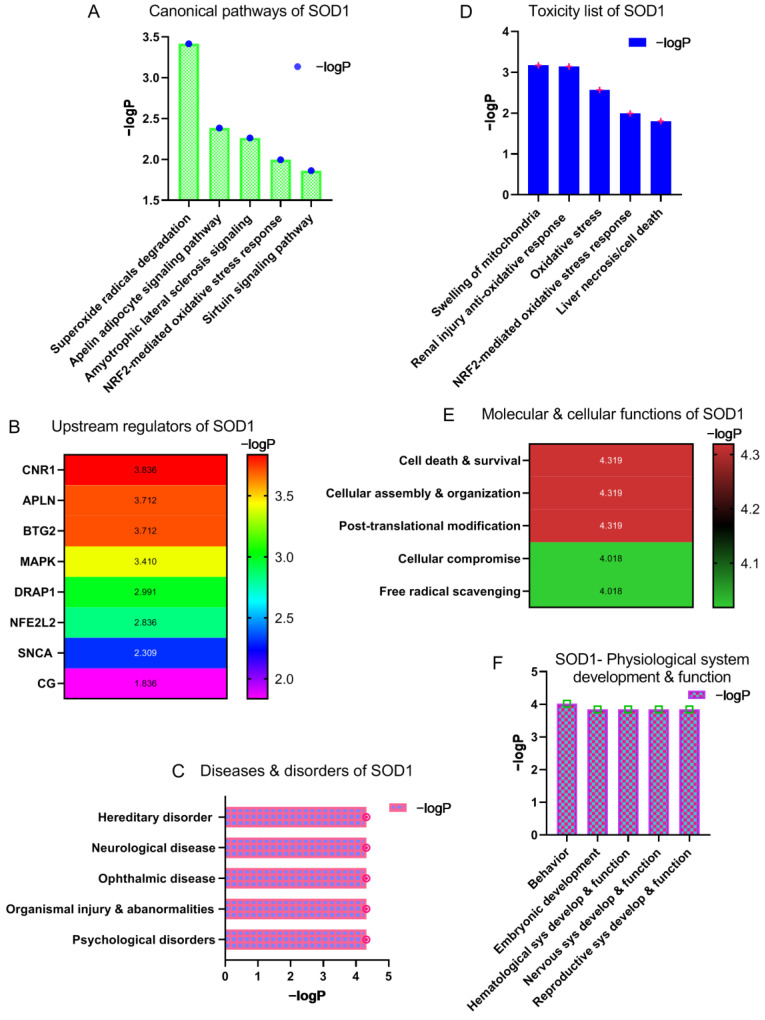
Canonical pathways, regulators, biological functions, role, and toxicity of SOD1. (**A**) Canonical signaling pathways of SOD1. (**B**) The upstream regulators of SOD1. (**C**) Diseases and disorders caused by abnormal expression of SOD1. (**D**) The list of toxicities caused by abnormal expression of SOD1. (**E**) Molecular and cellular functions of SOD1. (**F**) The role of SOD1 in physiological system development and its functions in organ systems. –logP indicates –log_10_(*p*-value), sys—system, develop—development.

IPA toxicity analysis of SOD1 identified superoxide radicals’ degradation as the leading signaling pathway followed by apelin adipocyte signaling, amyotrophic lateral sclerosis signaling, NRF2-mediated oxidative stress response, and sirtuin signaling pathways (Figure 2A). IPA toxicity analysis also revealed upstream regulators of SOD1. Cannabinoid receptor 1 (CNR1) was predicted to be the most important upstream regulator of SOD1. Additionally, IPA identified apelin (APLN), B-cell translocation gene 2 or BTG anti-proliferation factor 2 (BTG2), mitogen-activated protein kinase (MAPK), Dr1 associated protein 1 (DRAP1), nuclear factor erythroid 2-related factor 2 (NFE2L2), *α*-synuclein (SNCA), and cathepsin G (CG) as the upstream regulators of SOD1 (Figure 2B). In contrast to its protective antioxidant function, mutations in SOD1 result in brain diseases. According to the IPA analysis, hereditary disorders such as familial ALS are the leading outcome of SOD1-associated anomalous functions. Anomalies in SOD1 can also result in neurological diseases, ophthalmic diseases, organismal injuries and abnormalities, and psychological disorders. (Figure 2C). In accordance with SOD1’s involvement in a variety of diseases and disorders, our study found that SOD1 toxicity and response to it is manifested through mitochondrial swelling, renal injury associated with superoxide radicals, oxidative stress, NRF2-mediated oxidative stress response, and liver necrosis (Figure 2D). SOD1 performs a variety of molecular and cellular functions. Our study discovered that SOD1 plays a key role in cell death and survival, cellular assembly and organization, post-translation modification, cellular compromise, and free radical scavenging (Figure 2E). Additionally, SOD1 is involved in physiological system development and processes. IPA identified the role of SOD1 in behavior, embryonic development, hematological system development and functions, nervous system development and functions, and reproductive system development and functions (Figure 2F).

### 3.2. Identification of SOD1-ALS Pathway Molecules

**Figure 3:**
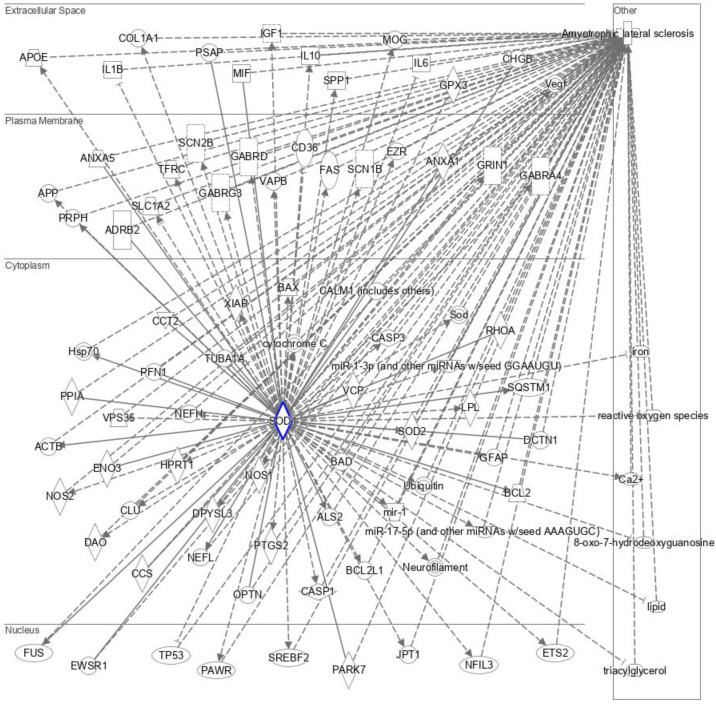
SOD1-ALS pathway molecules that are altered in response to SOD1 mutation and play a role in the development of amyotrophic lateral sclerosis, identified by IPA. Solid lines represent direct interaction, while dotted lines represent indirect interaction.

Mutation in SOD1 leads to the development of ALS. We used IPA to identify the molecules which are altered in response to SOD1 mutant-induced toxicity in ALS. IPA mapped 87 molecules which orchestrate a direct or indirect network between SOD1 and ALS (Figure 3). IPA identified PARK7 (Parkinson protein 7) as one of the key SOD1-ALS pathway molecules. PARK7 displayed a direct relationship with SOD1. Given the role of PARK7 as a sensor of oxidative stress, its critical relationship with SOD1 was not surprising. In addition to PARK7, IPA revealed several molecules that are central to protein aggregopathy as a part of the SOD1-ALS pathway, such as ALS2, FUS (ALS6; fusion gene), PFN1 (ALS18; profilin-1), APP (AD1; A*β* precursor protein), OPTN (ALS12; optineurin), and CLU (clusterin). The presence of these molecules in the SOD1–ALS pathway suggests that SOD1 inclusions precipitate the aggregation of other similar proteins, intensifying ALS symptoms.

IPA highlighted that impaired lipid homeostasis and metabolism plays a crucial role in the development of SOD1-triggered ALS. This was evident by the presence of several molecules related to lipid regulation and catabolism in the SOD1-ALS pathway, such as SREBF2 (sterol regulatory element binding transcription factor 2), APOE (apolipoprotein E), PSAP (prosaposin), and LPL (lipoprotein lipase).

SOD1-induced toxicity in ALS also alters neurotransmission in the brain. Various transport proteins and neurotransmitters, specifically those present in the plasma membrane such as SLC1A2 (solute carrier family 1 member 2), GABRG3 (gamma-aminobutyric acid type A receptor subunit gamma3), GABRD (gamma-aminobutyric acid type A receptor subunit delta), GRIN1 (glutamate receptor ionotropic, NMDA 1), GABRA4 (gamma-aminobutyric acid type A receptor subunit alpha4), SCN1B (sodium voltage-gated channel beta subunit 1), SCN2B (sodium voltage-gated channel beta subunit 2), VAPB (VAMP-associated protein B/C), and ANXA5 (annexin A5) appeared as members of the SOD1-ALS pathway.

SOD1 is abundantly present in the cytoplasm, where the SOD1 aggregates are bound to interact with molecules concerning cytoskeleton dynamics. IPA identified cytoplasm molecules regulating neuronal architecture, namely RHOA (Ras homolog family member A), DPYSL3 (dihydropyrimidinase-like 3), TUBA1A (tubulin alpha 1A), ACTB (B-actin), and NEFL (neurofilament light chain) as a part of SOD1-ALS pathway molecules. In addition to dysregulating cytoskeleton architectural constituents, SOD1 aggregates impair the function of traffic proteins. IPA showed that DCTN1 (dynactin 1) and VPS35 (vacuolar protein sorting ortholog 35), which are involved in the retrograde transport of proteins, are altered in response to SOD1 toxicity.

The overwhelming presence of inflammation and immune response factors, such as TP53 (tumor protein 53; nucleus), PAWR (pro-apoptotic WT1 regulator), BAX (BCL2 associated X protein), BAD (BCL2 associated agonist of cell death), BCL2 (B-cell lymphoma-2), CASP1 (caspase 1), CASP3 (caspase 3), PTGS2 (prostaglandin-endoperoxide synthase 2/COX2), NOS (nitric oxide synthase), XIAP (X-linked inhibitors of apoptosis), CD36 (cluster of differentiation 36), FAS (Fas cell surface death receptor/TNFRSF6), IL1B, IL6, IGF1 (insulin-like growth factor 1), VEGF (vascular endothelial growth factor A), GPX3 (glutathione peroxidase 3), SQSTM (sequestosome 1), SPP1 (secreted phosphoprotein 1), ANXA1 (annexin A1), and PPIA (peptidylprolyl isomerase A) in ALS-SOD1 pathway indicates that SOD1-induced ALS development is markedly associated with inflammation and immune response. Many of these inflammatory and immune response markers are known to activate ERK/MAPK signaling pathway.

### 3.3. GO Analysis of SOD1-ALS Pathway Genes

**Figure 4:**
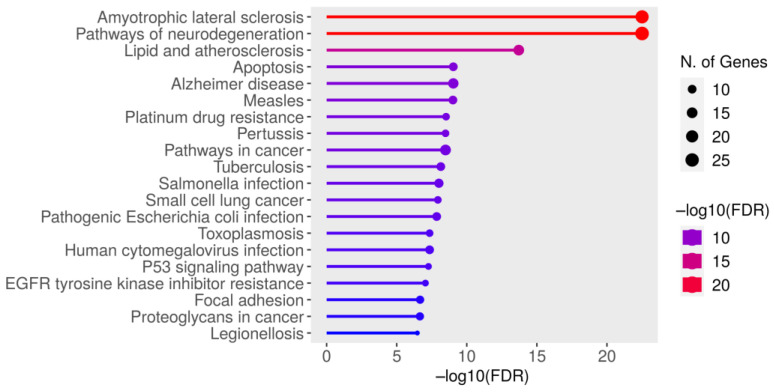
The GO analysis of IPA-predicted SOD1-ALS pathway molecules by ShinyGO. The GO analysis identified amyotrophic lateral sclerosis and neurodegeneration signaling as the key pathways.

IPA identified 87 molecules, including 75 genes associated with SOD1 toxicity, leading to the development of ALS (Figure 3; Table S2). These genes were further subjected to GO analysis to shed a light on the relationship between SOD1 and ALS and decipher the role of these molecules (genes) in the SOD1-ALS pathway. The GO analysis of SOD1-ALS network molecules unraveled that ALS and neurodegeneration pathways were significantly higher enriched than other pathways (Figure 4 and Figures S1–S3). The SOD1-ALS pathway molecules such as BAD, ALS2, NOS2, BAX, NOS1, FUS, OPTN, VAPB, PRPH, SOD1, CASP3, VCP (valosin-containing protein), TUBA1A, BCL2L1, BCL2, GRIN1, DCTN1, GPX3, and NEFL play a critical role in the development of SOD1-induced ALS (Figure S2) and neurodegeneration (Figure S3). Additionally, SOD1-ALS pathway molecules, such as ACTB, PFN1, SLC1A2, CASP1, and TP53 also play a part in the development of SOD1-provoked ALS (Figure S2), while FAS, PTGS2, PARK7, IL1B, IL6, APP, and CALM1 (calmodulin 1) engage in SOD1-triggered neurodegeneration in ALS (Figure S3).

The GO analysis also identified enriched biological processes (BP), molecular functions (MF), and cellular components (CC) of SOD1-ALS pathway molecules (Table 1). The GO analysis revealed response to chemical, regulation of biological quality, response to organic substance, positive regulation of transport, and signaling as the top-enriched biological processes of SOD1-ALS network molecules. The highest-enriched molecular functions associated with SOD1-ALS network molecules were identical protein binding, protein binding, protein domain specific binding, enzyme binding, and signaling receptor binding. The GO analysis identified vesicle, extracellular space, extracellular region, cell junction, and synapse as the most enriched cellular components of SOD1-ALS pathway molecules.

### 3.4. Construction and Analysis of Interaction Network of SOD1-ALS Pathway Molecules 

**Figure 5:**
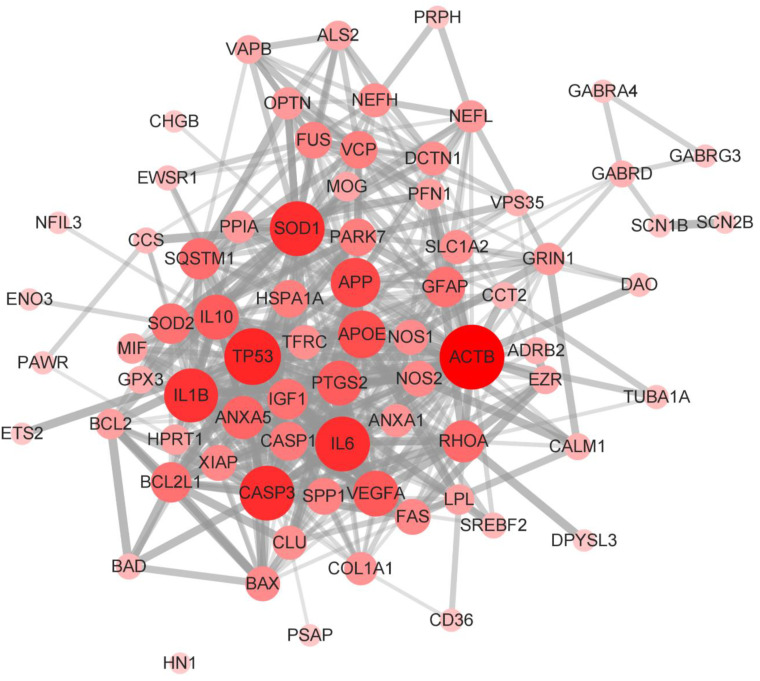
The molecular interaction network (PPI network) of IPA-identified SOD1-ALS pathway molecules. The PPI network was constructed using STRING and analyzed with Cytoscape. The Cytoscape network analysis predicted ACTB followed by TP53, IL6, CASP3, SOD1, IL1B, APP, APOE, and VEGFA as the major network hubs. The nodes’ size and color intensity correspond to the size of the network hub.

IPA-predicted SOD1-ALS pathway molecules (75 genes) were imported on the STRING server for the construction of a molecular interaction network (PPI network). The STRING-constructed interaction network displayed 75 nodes, 510 edges, 13.6 average node degree, 0.624 average local clustering coefficient, 174 expected number of edges, and PPI enrichment *p*-value of <1.0 × 10^−16^. The PPI network constructed on STRING was imported to Cytoscape for analysis. The Cytoscape network analysis revealed ACTB as the major network hub followed by TP53, IL6, CASP3, SOD1, IL1B, APP, APOE, VEGFA, IL10, PTGS2, ANXA5, RHOA, and SQSTM1 (Figure 5). The observance of ACTB as a prominent network hub was not surprising since SOD1 is mainly located in the cytoplasm and ACTB is a major component of the cytoskeleton. APP is a major neurodegenerative amyloid protein that likely contributes to the development and progression of ALS upon mutation in SOD1. APOE, besides being co-expressed with APP, mediates the clearance of lipoproteins. The dysregulation of APOE alters the lipid profile in the body, which may contribute to SOD1-induced ALS. The finding of inflammatory and immune response mediators as major network hubs is consistent with the marked presence of inflammatory and immune response mediators in the SOD1-ALS pathway identified by IPA.

## 4. Discussion

IPA QIAGEN is one of the leading tools for the analysis and interpretation of data acquired from omics experiments [21]. With IPA one can predict the expression, toxicity, metabolomics, and a variant effect analysis of molecule(s). SOD1 is an antioxidant enzyme, but mutations in SOD1 result in cellular stress and the progressive development of ALS. The number of SOD1 variants that trigger ALS continues to rise. This piqued our interest to extract more information about the role and toxicity of SOD1, with an emphasis on its toxicity in ALS. SOD1 detoxifies superoxide radicals into oxygen and hydrogen peroxide (Figure S4). Hydrogen peroxide is further broken down by catalase into water and oxygen. Apelin (APLN) is an adipocytokine. Under hypoxic conditions, hypoxia-inducible factor 1 subunit alpha (HIF1A) activates APLN, which in turn increases the activity of SOD1 via MAPK-ERK1/2 and AMPK axes (Figure S5). The mutated SOD1 is susceptible to misfolding, resulting in the formation of aggregate species in ALS (Figure S2). The misfolded SOD1 conformers have been implicated in the degeneration of spinal cord motor neurons (Figure S3). In addition, the misfolded SOD1 species not only impair normal SOD1 functions but also exhibit unusual interactions with other proteins, thus contributing to SOD1 toxicity via both loss and gain of functions [18]. NRF2, also known as nuclear factor erythroid 2-related factor 2, is a transcription factor, encoded by the NFE2L2 gene. Reactive oxygen species under oxidative stress activate NFE2L2 via RAS, MAP3K1/5/7, and PKC signaling. Following activation, NFE2L2 in the nucleus transcripts several genes, including SOD1 (Figure S6). However, under oxidative stress conditions, SOD1 oxidation leads to impaired dimer formation and misfolded proteins, resulting in the oligomerization and aggregate formation of SOD1. Sirtuin (SIRT5)-SOD1 interaction takes place in mitochondria, where under normal physiological conditions SIRT5 activates SOD1, leading to the detoxification of reactive oxygen species.

CNR1 was identified as the foremost regulator of SOD1 (Figure 2B). In the case of mutant SOD1-induced ALS, the expression of CNR1 increases while endocannabinoids accumulate in the spinal cord. This suggests that endocannabinoids play a protective role against neurodegeneration, and CNR1 might be a potential therapeutic target for ALS. The role of SOD1 regulator APLN was discussed earlier in the apelin adipocyte signaling pathway. Another regulator, BTG2, activates the antioxidant transcription factor NFE2L2, which in turn elevates the level of SOD1. Similar to BTG, MAPK also regulates SOD1 via the activation of NFE2L2. DRAP1 is a transcription repressor gene that is elevated in hypoxic conditions and likely governs SOD1 activity in conjunction with HIF1. SNCA, like SOD1, is an aggregate-forming protein, which potentiates SOD1 toxicity by accelerating SOD1 oligomerization. Similar to other upstream regulators, CG (CTSG) may also play a crucial role in oxidative stress.

Mutation in SOD1 compromises its functions, resulting in hereditary and neurological diseases including ALS (Figure 2C). SOD1 mutants tend to form aggregates that cause mitochondrial dysfunction, leading to neuronal cell death (Figure 2D). At the same time, oxidative stress promotes and potentiates SOD1 aggregation, resulting in exacerbated cell death. Furthermore, SOD1 regulates molecular and cellular functions such as cell death and survival via BCL2-BAX molecules, while it maintains cellular architecture via ACTN (Figure 2E). The post-translational modification of SOD1 causes misfolding of SOD1 in the cytoplasm and mitochondria. The misfolded protein in the mitochondria oligomerizes to form aggregates. The aggregated SOD1 loses its capacity to neutralize reactive oxygen species, impairing the electron transport chain (ETC). The cascade of events further leads to the release of mitochondrial cytochrome C through the formation of the BAX-BAK channel, which destroys mitochondrial homeostasis and results in caspase-mediated cell death (Figure S2) [18].

Since ALS was predicted to be the major outcome of SOD1 toxicity, we explored the pathway molecules between SOD1 toxicity and ALS. Our study discovered a network of molecules transmitting SOD1 toxicity, resulting in the development of ALS (Figure 3). Based on the IPA-identified SOD1-ALS pathway molecules, it can be assumed that SOD1-induced toxicity in ALS is accompanied by aggregation of other misfolded proteins, lipid dysregulation, impaired neurotransmission, compromised vesicular transport, and perturbed cytoskeleton dynamics. In addition to this, the plethora of immune and inflammatory mediators are altered during the course of SOD1-triggered ALS development. However, whether IPA-identified SOD1-ALS pathway molecules are altered due to explicit SOD1 toxicity or they are altered as a result of secondary non-specific or host stress response to SOD1 toxicity, remains to be discerned. The comparison of the molecular interaction network of wild-type SOD1 and mutant SOD1 could shed a light on this issue. Furthermore, SOD1 toxicity network molecules can be compared with the toxicity network of other amyloid proteins like APP, MAPT (tau), SNCA, and HTT (Huntingtin) to conclude which IPA-identified SOD1-ALS pathway molecules are exclusive to SOD1 toxicity only. We further subjected IPA-identified molecules to GO analysis, which ascertained the IPA finding that SOD1-ALS network molecules are most significantly associated with ALS and neurodegeneration signaling pathways (Figure 4; Figures S1–S3). The GO-enriched biological processes, such as response to chemical and response to organic substance, indicate the cellular response of SOD1-ALS network molecules against SOD1 toxicity, whereas regulation of biological quality highlights the up- or down-regulation of network molecules as a result of SOD1 toxicity (Table 1). The GO-enriched molecular functions, such as identical protein binding and protein binding, suggest the interaction of SOD1 homodimer with network molecules and the binding of network molecules with the interacting proteins, respectively (Table 1). The GO-enriched top-ranked cellular component vesicle points to the association of network molecules with apoptotic bodies and cytoplasmic vesicles (Table 1).

To analyze the interactions among IPA-predicted SOD1-ALS pathway molecules, we constructed a PPI network using STRING, which was analyzed with Cytoscape (Figure 5). The appearance of cytoplasmic ACTB as the largest network hub is understandable since it regulates neuronal cell growth and motility and preserves cellular architecture. SOD1, APP, and APOE drive amyloid pathology in the brain and spinal cord, while amyloidosis-resulted neuroinflammation and cell death are mediated by TP53, IL6, CASP3, IL1B, and VEGFA.

## 5. Conclusions

In light of the growing number of SOD1 variants causing ALS, researchers are still struggling to understand the complex SOD1 interaction network at work in ALS and neurodegeneration. In this study we used an integrated bioinformatics approach to analyze the role and function of SOD1, with an emphasis on uncovering SOD1 network molecules in ALS. Our study identified signaling pathways, regulators, and molecular interaction network of SOD1 that dominates ALS. Researchers in the wet lab can build upon our findings to elucidate the mechanism by which SOD1 mutants cause amyloid pathology.

## Figures and Tables

**Table 1:** The GO enrichment analysis of SOD1-ALS pathway molecules, highlighting top-enriched biological processes, molecular functions, and cellular components.

	Description (Term)	Gene Counts	*p*-Value
**BP**	Response to chemical (GO:0042221)	55	7.84 × 10^−23^
	Regulation of biological quality (GO:0065008)	53	3.34 × 10^−22^
	Response to organic substance (GO:0010033)	47	8.62 × 10^−22^
	Positive regulation of transport (GO:0051050)	29	4.89 × 10^−20^
	Signaling (GO:0023052)	56	1.02 × 10^−19^
**MF**	Identical protein binding (GO:0042802)	38	2.88 × 10^−20^
	Protein binding (GO:0005515)	62	4.54 × 10^−19^
	Protein domain specific binding (GO:0019904)	18	8.00 × 10^−11^
	Enzyme binding (GO:0019899)	29	2.83 × 10^−10^
	Signaling receptor binding (GO:0005102)	23	4.65 × 10^−09^
**CC**	Vesicle (GO:0031982)	43	7.79 × 10^−14^
	Extracellular space (GO:0005615)	37	5.07 × 10^−12^
	Extracellular region (GO:0005576)	41	2.86 × 10^−11^
	Cell junction (GO:0030054)	28	2.63 × 10^−10^
	Synapse (GO:0045202)	23	2.30 × 10^−10^

## Data Availability

Not applicable.

## Supplementary Materials

The following supporting information can be downloaded at: https://www.mdpi.com/article/10.3390/brainsci13010151/s1, Figure S1: Interactive pathway plot for SOD1-ALS network molecules predicted by ShinyGO analysis, Figure S2: Amyotrophic lateral sclerosis pathway, Figure S3: Neurodegeneration pathway, Figure S4: Superoxide radicals’ degradation by SOD1, Figure S5: The role of SOD1 in the apelin adipocyte signaling pathway, Figure S6: (**A**) NRF2-mediated oxidative stress response signaling pathway in the extracellular space and cytoplasm. (**B**) The role of SOD1 in the NRF2-mediated oxidative stress response signaling pathway in the nucleus; Table S1: SOD1 variants causing amyotrophic lateral sclerosis, Table S2: The list of SOD1-ALS pathway molecules identified by IPA, genes used for ShinyGO analysis, and construction of a molecular interaction network on STRING.
