# Supplementary material for: The Role of Superoxide Dismutase 1 in Amyotrophic Lateral Sclerosis: Identification of Signaling Pathways, Regulators, Molecular Interaction Networks, and Biological Functions through Bioinformatics
Source: Brain Sci. 2023 Jan 15;13(1):151. doi: 10.3390/brainsci13010151 (PMC9857031; doi:10.3390/brainsci13010151)
Supplement: Supplementary file 1 [file brainsci-13-00151-s001.zip › brainsci-2094474-supplementary.pdf]

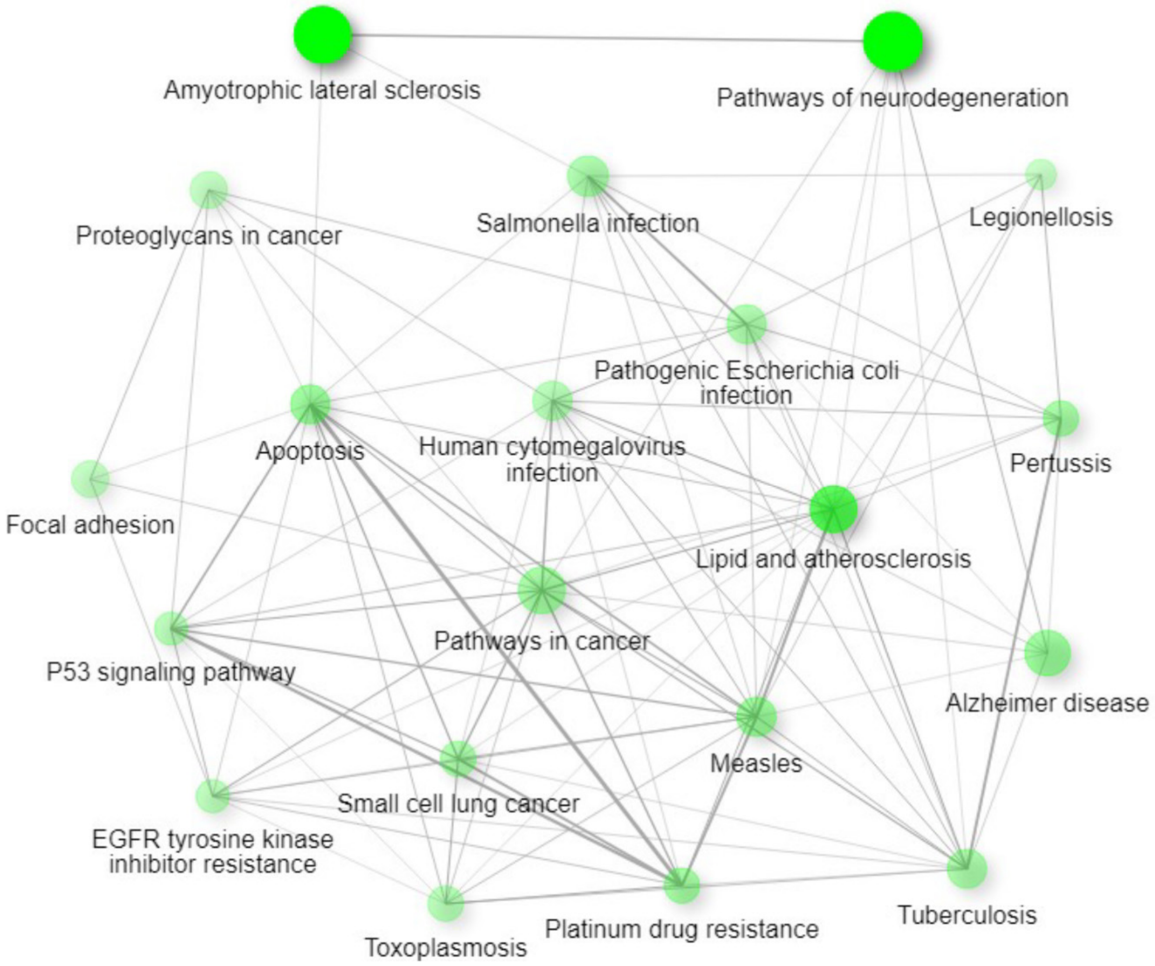


**Figure S1.** Interactive pathway plot for SOD1–ALS network molecules predicted by ShinyGO analysis. Two pathways were considered connected if they shared ≥20% genes. Intense green nodes represent highly enriched gene datasets while bigger nodes represent large gene datasets. The thicker lines indicate higher overlapping of genes.

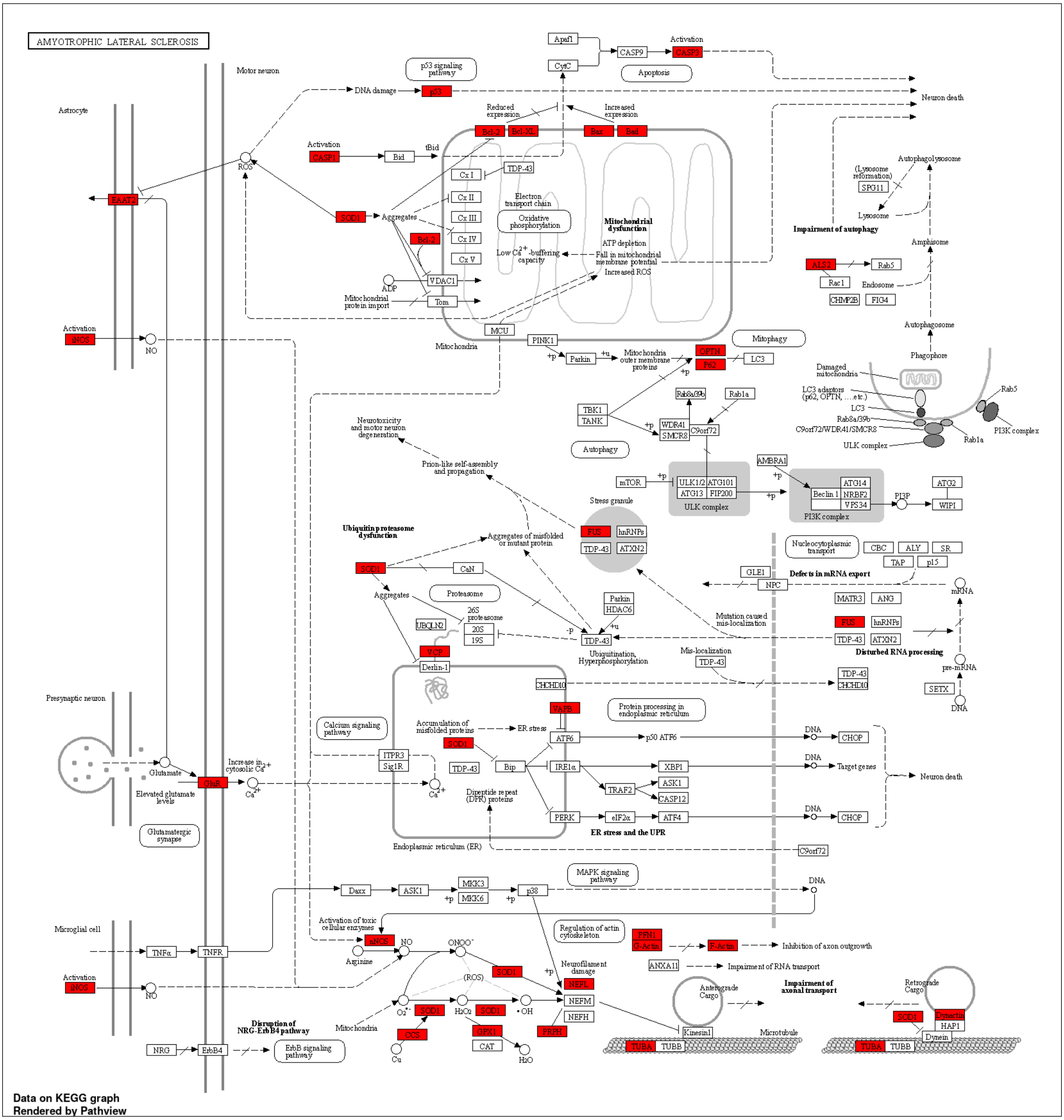


**Figure S2.** Amyotrophic lateral sclerosis pathway. The SOD1–ALS pathway molecules predicted by IPA are highlighted in red. The molecule boxes were painted red with ShinyGO. Figure source KEGG graph, rendered by Pathview.

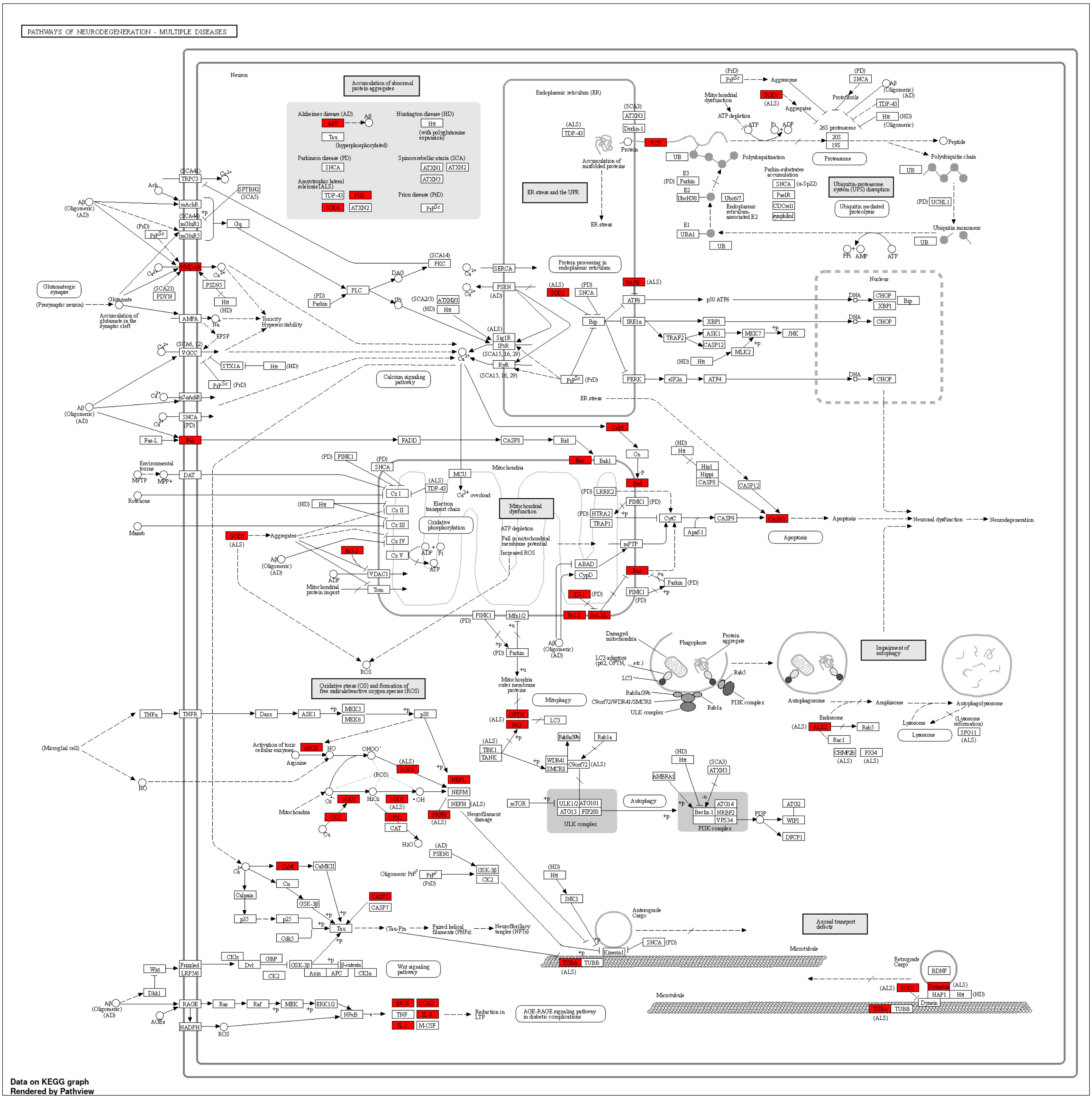


**Figure S3.** Neurodegeneration pathway. The SOD1–ALS pathway molecules predicted by IPA are highlighted in red. The molecule boxes were painted red with ShinyGO. Figure source KEGG graph, rendered by Pathview.

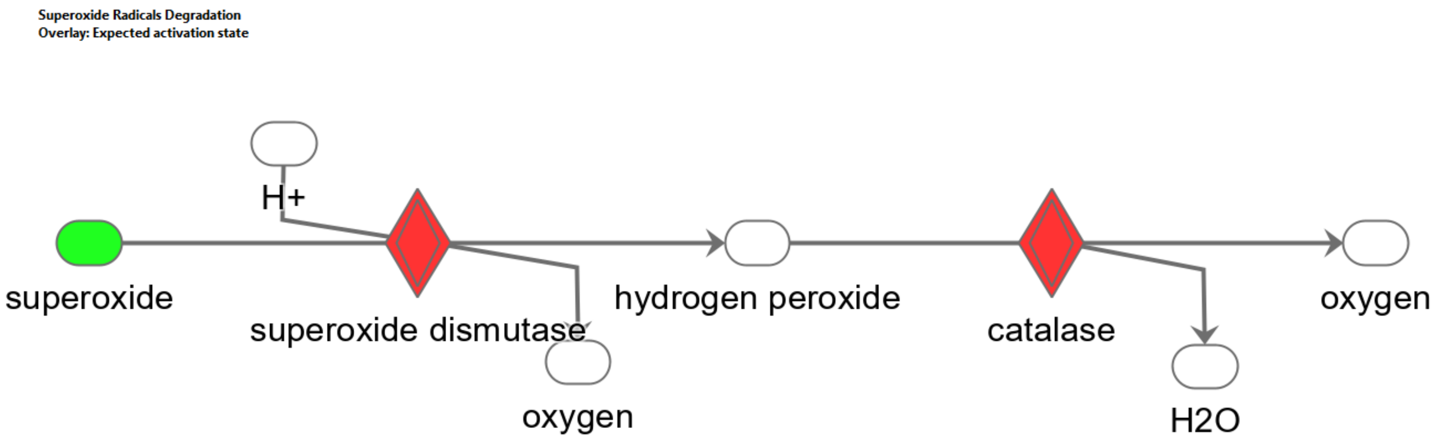


**Figure S4.** Superoxide radicals' degradation by SOD1. Figure adapted from IPA, QIAGEN.

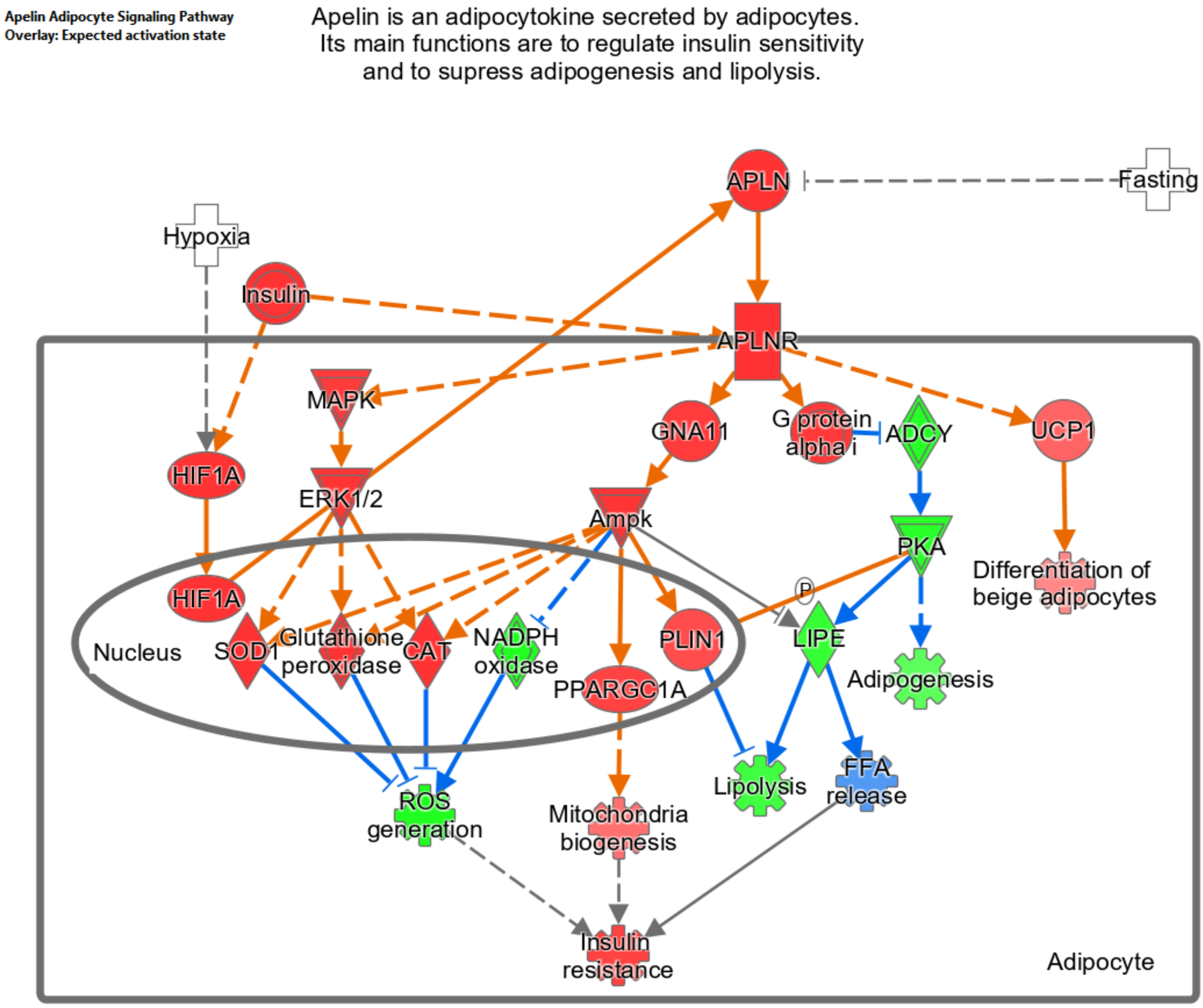


**Figure S5.** The role of SOD1 in the apelin adipocyte signaling pathway. Figure adapted from IPA, QIAGEN.

**A)**

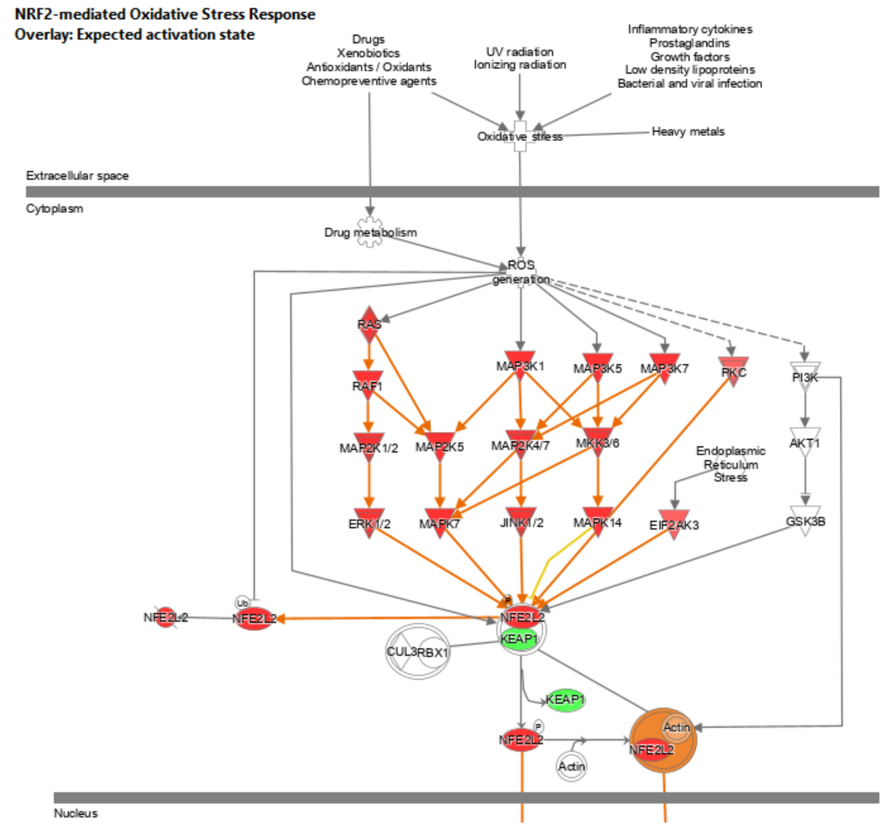
NRF2-mediated Oxidative Stress Response
Overlay: Expected activation state
Drugs
Xenobiotics
Antioxidants / Oxidants
Chemopreventive agents
UV radiation
Ionizing radiation
Inflammatory cytokines
Prostaglandins
Growth factors
Low density lipoproteins
Bacterial and viral infection
Oxidative stress
Heavy metals
Extracellular space
Cytoplasm
Drug metabolism
ROS generation
RAS
RAF1
MAP3K1
MAP3K5
MAP3K7
PKC
PI3K
MAP2K1/2
MAP2K5
MAP2K4/7
MKK3/6
AKT1
Endoplasmic Reticulum Stress
ERK1/2
MAPK7
JNK1/2
MAPK14
EIF2AK3
GSK3B
NFE2L2
Ub
NFE2L2
P
NFE2L2
KEAP1
CUL3
RBX1
KEAP1
P
NFE2L2
Actin
Actin
NFE2L2
Nucleus

**B)**

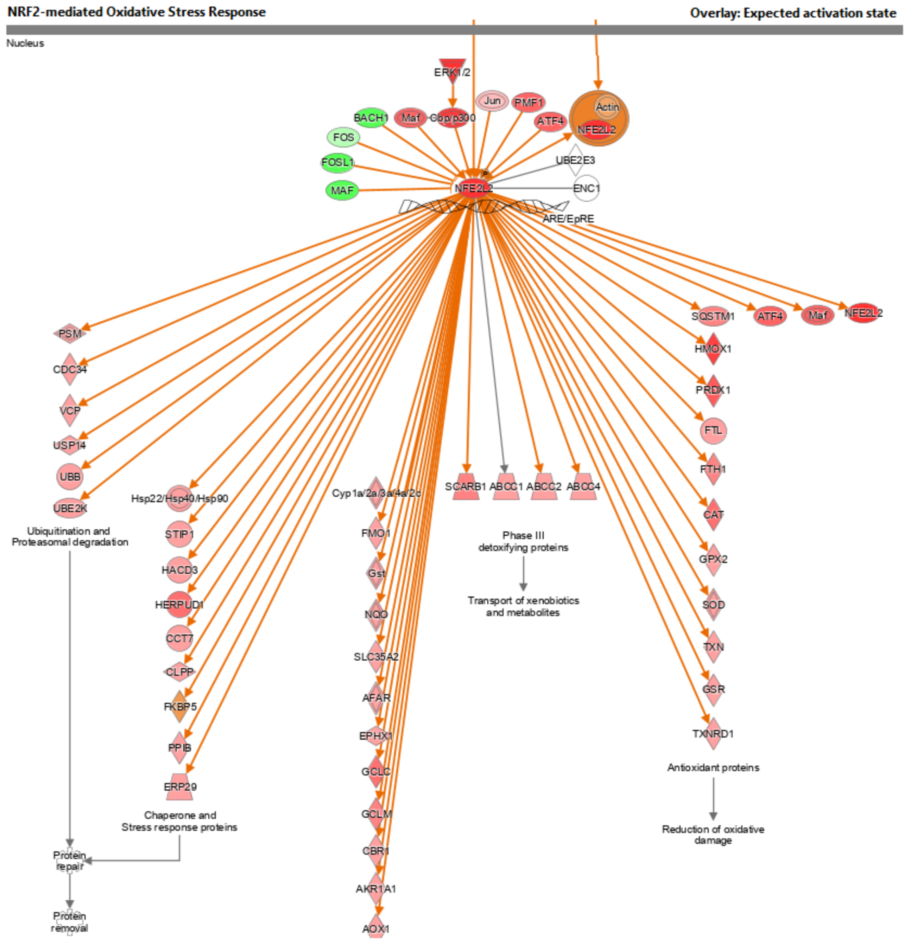
NRF2-mediated Oxidative Stress Response
Overlay: Expected activation state
Nucleus
ERK1/2
Jun
PMF1
BACH1
Maf
Cbp/p300
ATF4
Actin
NFE2L2
FOS
FOSL1
UBE2E3
MAF
P
NFE2L2
ENC1
ARE/EpRE
SQSTM1
ATF4
Maf
NFE2L2
PSM
CDC34
VCP
USP14
UBB
UBE2K
Ubiquitination and Proteasomal degradation
Hsp22/Hsp40/Hsp90
STIP1
HACD3
HERPUD1
CCT7
CLPP
FKBP5
PPIB
ERP29
Chaperone and Stress response proteins
Cyp1a/2a/3a/4a/2c
FMO1
Gst
NQO
SLC35A2
AFAR
EPHX1
GCLC
GCLM
CBR1
AKR1A1
AOX1
SCARB1
ABCC1
ABCC2
ABCC4
Phase III detoxifying proteins
Transport of xenobiotics and metabolites
HMOX1
PRDX1
FTL
FTH1
CAT
GPX2
SOD
TXN
GSR
TXNRD1
Antioxidant proteins
Reduction of oxidative damage
Protein repair
Protein removal

**Figure S6. A)** NRF2-mediated oxidative stress response signaling pathway in the extracellular space and cytoplasm. Figure adapted from IPA, QIAGEN. **B)** The role of SOD1 in the NRF2-mediated oxidative stress response signaling pathway in the nucleus. Figure credit to IPA, QIAGEN.

**Table S1.** SOD1 variants causing amyotrophic lateral sclerosis.

| | | | | | |
|---|---|---|---|---|---|
| A141A | D12Y | G11G | H121L | L68P | T55R |
| A141G | D77V | G11R | H44R | L68R | T89_D91del |
| A146G | D77Y | G11V | H47D | L85F | V119KfsTer5 |
| A146T | D84G | G128R | H47R | L85V | V119L |
| A5F | D84N | G128VfsTer7 | H49Q | L9Q | V119M |
| A5S | D91A | G139E | H49R | L9V | V120F |
| A5T | D91A | G13R | H72Y | N139H | V149A |
| A5V | D91N | G142A | H81R | N140D | V149G |
| A90T | D91V | G142E | I100V | N140K | V15G |
| A90V | D91_K92insN | G142R | I105F | N140N | V15M |
| A96T | D97MfsTer8 | G142X | I113M | N20S | V30A |
| c.170-108T>A | D97N | G148 | I113T | N66S | V30Dfs*8 |
| c.239+62T>C | E101G | G148C | I114F | N87D | V30M |
| c.240-7T>G | E101K | G148D | I114T | N87I | V32A |
| c.358-10A>G | E122G | G148R | I150T | N87K | V48A |
| c.358-10T>G | E133DfsTer2 | G16A | I150V | N87S | V48F |
| c.358-11A>G | E133insTT | G17A | I152S | P67R | V6L |
| c.358-304C>G | E133K | G28_P29del | I152T | P67S | V88A |
| C112Y | E133VfsX31 | G38R | I19del | Q154Q | V88M |
| C147R | E134del | G38V | K129PfsX6 | Q22H | V8E |
| C147X | E134K | G42D | K129WfsX6 | Q23L | V95A |
| C7F | E134V | G42S | K137X | Q23R | V98L |
| C7G | E22G | G62R | K4E | R116C | V98M |
| C7S | E22K | G73C | K92RfsX9 | R116G | |
| C7W | E41G | G73D | L106V | S105del | |
| C7Y | E50K | G73S | L107F | S106L | |
| D102G | F21C | G86R | L118V | S108LfsX15 | |
| D102H | F21L | G86S | L127GfsTer6 | S135N | |
| D102N | F46C | G94A | L127S | S135T | |
| D102Y | F46S | G94C | L127X | S60I | |
| D110Y | F65L | G94D | L145F | S60S | |
| D125G | G109V | G94R | L145S | T117T | |
| D125V | G10A | G94S | L39R | T138A | |
| D126H | G115A | G94V | L39V | T138R | |

Reference: ALSoD – Amyotrophic Lateral Sclerosis online Database. Available at: https://alsod.ac.uk/output/gene.php/SOD1 (accessed on 10 November 2022).

**Table S2.** The list of SOD1–ALS pathway molecules identified by IPA, genes used for ShinyGO analysis, and construction of a molecular interaction network on STRING.

| No | SOD1–ALS pathway molecules | Genes used for ShinyGO analysis | Genes used for STRING molecular interaction network construction |
|---|---|---|---|

| | | | |
|---|---|---|---|
| 1 | 8-oxo-7-hydrodeoxyguano-sine | ACTB | ACTB |
| 2 | ACTB | ADRB2 | ADRB2 |
| 3 | ADRB2 | ALS2 | ALS2 |
| 4 | ALS2 | ANXA1 | ANXA1 |
| 5 | ANXA1 | ANXA5 | ANXA5 |
| 6 | ANXA5 | APOE | APOE |
| 7 | APOE | APP | APP |
| 8 | APP | BAD | BAD |
| 9 | BAD | BAX | BAX |
| 10 | BAX | BCL2 | BCL2 |
| 11 | BCL2 | BCL2L1 | BCL2L1 |
| 12 | BCL2L1 | CALM1 | CALM1 |
| 13 | $Ca^{2+}$ | CASP1 | CASP1 |
| 14 | CALM1 (includes others) | CASP3 | CASP3 |
| 15 | CASP1 | CCS | CCS |
| 16 | CASP3 | CCT2 | CCT2 |
| 17 | CCS | CD36 | CD36 |
| 18 | CCT2 | CHGB | CHGB |
| 19 | CD36 | CLU | CLU |
| 20 | CHGB | COL1A1 | COL1A1 |
| 21 | CLU | DAO | DAO |
| 22 | COL1A1 | DCTN1 | DCTN1 |
| 23 | Cytochrome C | DPYSL3 | DPYSL3 |
| 24 | DAO | ENO3 | ENO3 |
| 25 | DCTN1 | ETS2 | ETS2 |
| 26 | DPYSL3 | EWSR1 | EWSR1 |
| 27 | ENO3 | EZR | EZR |
| 28 | ETS2 | FAS | FAS |
| 29 | EWSR1 | FUS | FUS |
| 30 | EZR | GABRA4 | GABRA4 |
| 31 | FAS | GABRD | GABRD |
| 32 | FUS | GABRG3 | GABRG3 |
| 33 | GABRA4 | GFAP | GFAP |
| 34 | GABRD | GPX3 | GPX3 |
| 35 | GABRG3 | GRIN1 | GRIN1 |
| 36 | GFAP | HPRT1 | HPRT1 |
| 37 | GPX3 | HSPA1A | HSPA1A |
| 38 | GRIN1 | IGF1 | IGF1 |
| 39 | HPRT1 | IL6 | IL6 |
| 40 | Hsp70 | IL10 | IL10 |
| 41 | IGF1 | IL1B | IL1B |
| 42 | IL6 | JPT1 | HN1 |
| 43 | IL10 | LPL | LPL |
| 44 | IL1B | MIF | MIF |
| 45 | Iron | MOG | MOG |
| 46 | JPT1 | NEFH* | NEFH |
| 47 | Lipid | NEFL | NEFL |
| 48 | LPL | NFIL3 | NFIL3 |
| 49 | MIF | NOS1 | NOS1 |
| 50 | mir-1 | NOS2 | NOS2 |
| 51 | miR-1-3p | OPTN | OPTN |
| 52 | miR-17-5p | PARK7 | PARK7 |

| | | | |
|---|---|---|---|
| 53 | MOG | PAWR | PAWR |
| 54 | NEFH | PFN1 | PFN1 |
| 55 | NEFL | PPIA | PPIA |
| 56 | Neurofilament | PRPH | PRPH |
| 57 | NFIL3 | PSAP | PSAP |
| 58 | NOS1 | PTGS2 | PTGS2 |
| 59 | NOS2 | RHOA | RHOA |
| 60 | OPTN | SCN1B | SCN1B |
| 61 | PARK7 | SCN2B | SCN2B |
| 62 | PAWR | SLC1A2 | SLC1A2 |
| 63 | PFN1 | SOD1 | SOD1 |
| 64 | PPIA | SOD2 | SOD2 |
| 65 | PRPH | SPP1 | SPP1 |
| 66 | PSAP | SQSTM1 | SQSTM1 |
| 67 | PTGS2 | SREBF2 | SREBF2 |
| 68 | Reactive oxygen species | TFRC | TFRC |
| 69 | RHOA | TP53 | TP53 |
| 70 | SCN1B | TUBA1A | TUBA1A |
| 71 | SCN2B | VAPB | VAPB |
| 72 | SLC1A2 | VCP | VCP |
| 73 | SOD1 | VEGFA | VEGFA |
| 74 | SOD2 | VPS35 | VPS35 |
| 75 | Sod | XIAP | XIAP |
| 76 | SPP1 | | |
| 77 | SQSTM1 | | |
| 78 | SREBF2 | | |
| 79 | TFRC | | |
| 80 | TP53 | | |
| 81 | Triacylglycerol | | |
| 82 | TUBA1A | | |
| 83 | Ubiquitin | | |
| 84 | VAPB | | |
| 85 | VCP | | |
| 86 | Vegf | | |
| 87 | VPS35 | | |
| 88 | XIAP | | |
| | | | |

*Not mapped in ShinyGO analysis, HSPA1A was mapped for Hsp70, blue color indicates that these molecules do not represent genes.
